# Validation of the Arabic version of the 35-item TEMPS-M in a community sample of adults

**DOI:** 10.1186/s40359-023-01064-y

**Published:** 2023-01-28

**Authors:** Feten Fekih-Romdhane, Ecem Yakın, Zeinab Bitar, Diana Malaeb, Toni Sawma, Sahar Obeid, Souheil Hallit

**Affiliations:** 1 Department of Psychiatry “Ibn Omrane”,The Tunisian Center of Early Intervention in Psychosis, Razi Hospital, 2010 Manouba, Tunisia; 2 Faculty of Medicine of Tunis, Tunis El Manar University, Tunis, Tunisia; 3 Centre d’Etudes Et de Recherches en Psychopathologie Et Psychologie de La Santé, Université de Toulouse-Jean Jaurès, UT2J, 5 Allées Antonio Machado, 31058 Toulouse, France; 4 Faculty of Medicine, Paris-Saclay University, Le Kremlin-Bicêtre, France; 5 College of Pharmacy, Gulf Medical University, Ajman, United Arab Emirates; 6 School of Pharmacy, Lebanese International University, Beirut, Lebanon; 7 Social and Education Sciences Department, School of Arts and Sciences, Lebanese American University, Jbeil, Lebanon; 8 Psychology Department, College of Humanities, Effat University, Jeddah, 21478 Saudi Arabia; 9 School of Medicine and Medical Sciences, Holy Spirit University of Kaslik, P.O. Box 446, Jounieh, Lebanon; 10 Applied Science Research Center, Applied Science Private University, Amman, Jordan; 11 Research Department, Psychiatric Hospital of the Cross, Jal Eddib, Lebanon

**Keywords:** Affective temperament, TEMPS-M, Arabic, Validation, Confirmatory factor analysis

## Abstract

**Background:**

To our knowledge, no brief version of the Temperament Evaluation in Memphis Pisa and San Diego (TEMPS-M) is available so far in the Arabic language, which might have resulted in limited research in this field from Arab countries. We aimed through this study to validate the 35-item TEMPS-M into the Arabic language in a sample of non-clinical Lebanese adults.

**Methods:**

We used an online cross-sectional survey targeting non-clinical Lebanese adults from the general population. A confirmatory factor analysis was conducted to test the factorial structure of the TEMPS-M.

**Results:**

*A*ll five temperament subscales achieved good/very good internal consistencies in the present study (depressive: α = 0.78, cyclothymic: α = 0.86, hyperthymic: α = 0.83, irritable: α = 0.87, and anxious: α = 0.87). The five-factor solution of the TEMPS-M displayed a good CFI of 0.94, TLI of .94 and a GFI of .95, but a poor RMSEA of .10 [90% CI .10, .11]. The five affective temperaments showed positive correlations with personality dysfunction domains, thus attesting for convergent validity. In addition, positive correlations between all affective temperament dimensions and anxiety/depression scores were found. We also tested for gender invariance, and found that configural, metric, and scalar invariance were supported across gender.

**Conclusion:**

Our data suggest that the psychometric properties of the Arabic TEMPS-M are good. Making this scale available in Arabic will hopefully encourage Arab researchers to investigate this under-explored topic in their countries, and advance knowledge on how culture impacts the prevalence, development and course of temperament.

## Background

Temperament represents a biological, genetically inherited and stable core of the personality that does not change throughout life; temperament helps in identifying a person’s basic level of reactivity, mood, and energy [1]. At first, Hippocrates and Aristotle introduced the human personality as a mix of temperamental categories; they believed that temperament represents a constitutional form of emotional reactivity [2]. After that, Kraepelin developed a theory consisting of 4 types of temperament (i.e. manic, depressive, cyclothymic and irritable), defined as the subclinical forms of affective disorders [3]. In 1958, Kurt Schneider provided excellent descriptions of the Kraepelinian theory and changed the term “manic” to “hyperthymic” [4].

Hans Eysenck [5, 6], Jerome Kagan [7, 8], Robert Cloninger [9, 10], and Hagop Akiskal [11, 12] all developed empirical theories of temperament and character traits and dimensions. Hans Eysenck (1916–1997) was the first to analyze personality differences using an empirical/statistical method. He proposed that the basic factors were Neuroticism (tendency to experience negative emotions), Extraversion (tendency to enjoy positive events) and Psychotisism (cognitive style). Eysenck’s theory and all the theories that derived from it, concern approach/reward, inhibition/punishment, and aggression/flight.

By adapting Schneider classic description combined with Kraepelin theory, Akiskal and his colleagues [12] were the first to develop a measurement tool called the TEMPS-I (The Temperament Evaluation of Memphis, Pisa, Paris and San Diego), consisting of a semi-structured interview and used to evaluate temperament traits. In 2005 and out of TEMPS-I, Akiksal and his colleagues [11, 13], developed TEMPS-A (Temperament Evaluation of Memphis, Pisa, Paris and San Diego Auto-questionnaire), but this version was a self-reported tool containing 110 items assessing the 5 temperament categories: depressive, cyclothymic, hyperthymic, irritable and anxious temperaments. Depressive temperament is when people tend to be self-denying and dedicate themselves to others. These people feel most satisfied when they are confronting to social norms [14]. They are usually the most vulnerable to clinical depression [15]. On the other hand, hyperthymic temperament is characterized by being extra confident, extra energetic, overoptimistic and in general having leadership traits [16] Irritable temperament comes as a subtype of cyclothymic temperament and is characterized by being more critical, dissatisfied, angry and complaining [17].Finally, anxious temperament is characterized by a fearful and exaggerated worrying [13].

This long version (TEMPS-A) was translated and validated in approximately 25 different languages i.e. Chinese [18], Italian [19], Serbian [20], Japanese [21], Hungarian [22], Polish [23], Turkish [24], French [25], and Arabic [26].

Although being psychometrically robust and the most widely used measure of affective temperaments, and despite its valuable contribution to our understanding of temperament structure and dynamics, this 110-item scale may impose a burden on respondents and be of limited clinical and research utility due to its length. Indeed, there is evidence that excessively long scales affect data quality [27], and often lead to les time spent answering each question, and less willingness to complete all questions [28]. Therefore, being too long and time-consuming makes the TEMPS-A not suitable for quick assessments and monitoring in clinical practice, or inclusion in large questionnaires with other measures for research purposes.

In order to address these gaps, shortened versions of the TEMPS-A have been developed, validated, and adapted for use in specific countries and cultures, such as the Portuguese version of the 45-item TEMPS-Rio de Janeiro [29], the TEMPS-A-39 (English [13], Italian [19], and Chinese [30] versions), and the TEMPS-M-35 (Münster-German cl [31]) and Austrian [32] versions). Besides being shorter, easier to use and more convenient for participants, the latter version (i.e., TEMPS-M) brings another important improvement relative to the original version which is the change in scoring format (from dichotomous yes/no responses to a five-point Likert scale) [31]. This change is likely to ameliorate the scale utility, enabling a multidimensional examination of the temperament construct [33].

However, to our knowledge, no brief version of the TEMPS is available so far in the Arabic language. This might have resulted in limited research in this field from Arab countries; we could find only a few studies conducted on this topic, mainly in Lebanon [34–38] and Tunisia [39–42]. Hence, the strong need for a psychometrically valid short version for Arabic-speaking populations. Adding to this idea, we decided to validate the TEMPS-M-35 rather than the 39 and 45-item as longer scales with more items imply higher cost of public health surveying [43]. Thus, using the shortest version would allow for a faster, easier to perform, more convenient, and lower cost assessing of temperaments in Arab settings. We believe that validating the 35-item TEMPS-M into the Arabic language would allow not only avoidance of unnecessary time and efforts on Arabic-speaking respondents, but also reduction of costs to clinicians and researchers from the Arab developing (lower-middle income) countries; while retaining the validity and reliability of the Arabic TEMPS-A. We therefore performed this research work to validate the TEMPS-M in a community‐derived sample of nonclinical Lebanese adults. Our main objectives were to (1) evaluate the reliability, convergent validity (as tested by correlating temperament dimensions with personality traits) of the Arabic 35-item TEMPS-M, and (2) examine the internal structure and measurement invariance by gender using confirmatory factor analysis.

## Methods

### Procedure and participants

Lebanese participants were recruited via a snowball technique, using a link created on Google forms. The research team approached participants they know at first, who were solicited to send the link to other family members and friends. Inclusion criteria for study participation were (a) being 18 years of age and above, (b) to have a minimal level of literacy (to read Arabic and write). Subjects participated voluntarily and provided informed consent prior to data collection (via button click to the first question in the online survey). There was no compensation in return for participation.

### Minimum sample size

A previous study suggested that the minimum sample size to conduct a confirmatory factor analysis ranges from 3 to 20 times the number of the scale’s variables [44]. Therefore, we assumed a minimum sample of 350 participants needed to have enough statistical power based on a ratio of 10 participants per one item of the scale.

### Measures

*Temperament* Affective temperament traits were assessed using the Arabic version [31] of the Temperament Evaluation of Memphis, Pisa, Paris, and San Diego (TEMPS). It included 35 items, scored on a 5-point Likert scale ranging from 1 (not at all) to 5 (very much). The 35 self-rating items can be assigned to five subscales: depressive (i.e. tending towards rigid thinking, self-accusation, and shyness), cyclothymic (i.e. being moody and changeable, tending towards superficial thinking and intense emotion), hyperthymic (i.e. being strongly extroverted and expansive), irritable (i.e. showing higher energy and anger, but on the other hand a lower level of empathy, and dissatisfaction), and anxious (i.e. tending towards worry, ruminate, and continuous tension). Subscale scores range from 5 to 35, with higher scores denoting higher expressions of the temperament.

*Personality inventory for DSM-5—Brief Form (PID-5-BF)* This scale is composed of 25 items, rated on a scale from 0 (very false or often false) to 3 (very true or often true) [45]. Five scores derive from this scale as follows: negative affect (α = 0.68), detachment (α = 0.70), antagonism (α = 0.71), disinhibition (α = 0.80) and psychoticism (α = 0.70). Higher scores indicate greater personality dysfunction in each domain.

*Hamilton Anxiety Rating Scale* Validated in Lebanon [46] this scale is composed of 14 items, rated on a five-point Likert scale. Higher scores reflect higher anxiety (α = 0.93).

*Hamilton Depression Rating Scale*. Validated in Lebanon [47], this scale is composed of 17 items, with higher scores reflect higher depression (α = 0.88).

*Sociodemographics* The survey was complemented by the collection of the participant’s age (years), gender (male, female), marital status (married, not married), and educational level.

### Translation procedure

The scales (TEMPS and PID-5-BF) were first translated from English to Arabic by one psychologist familiar with the scales’ concepts. Her mother tongue was Arabic and fluent in English. The Arabic version was verified by a linguistic professional. A committee composed of the research team, one psychiatrist, one psychologist and the translator verified the conceptual equivalence of the Arabic version [ref]. The Arabic version of the scales was back translated to English by another psychologist, fluent in both English and Arabic as well. The committee members compared both English versions to discern any discrepancies; all procedures were done according to the international recommendations of forward-back translation [48]. A pilot test was done on thirty participants enrolled through convenient sampling to ensure that all questions were well understood. The responses collected during the pilot test were not included in the final database.

### Statistical analysis

A five-factor confirmatory analysis was conducted to test the factorial structure of the TEMPS. We used Weighted Least Squares with Mean and Variance (WLSMV) estimation method which is more appropriate for ordinal data. Confirmatory factor analysis was conducted in RStudio (Version 1.4.1103 for Macintosh), using the Lavaan and semTools packages. In fact, values greater than 0.90 and 0.95 for the CFI and TLI, values closer to 1.00 for the GFI indicate a better model fit [49, 50]. However, values for the RMSEA are expected to be at or below 0.08 to represent a good model fit [49, 50].

To examine gender invariance of the TEMPS-M scores, we conducted multi-group CFA [51]. Measurement invariance was assessed at the configural, metric, and scalar levels [52]. Following the recommendations of Cheung and Rensvold (2002) and Chen (2007), we accepted ΔCFI ≤ 0.010 and ΔRMSEA ≤ 0.015 or ΔSRMR ≤ 0.010 (0.030 for factorial invariance) as evidence of invariance. We aimed to test for gender differences on latent FAS scores using an independent-samples *t*-test only if scalar or partial scalar invariance were established.

Missing data constituted less than 5%, thus, was not replaced. To assess reliability, Cronbach’s α values were computed for each subscale and scale. Cronbach’s α values of ≥ 0.70 were considered acceptable. Finally, we examined the skewness and kurtosis values for the temperament subscales scores, which were within defined range (skewness and kurtosis between − 1 and + 1; [53]). Therefore, the sample was considered normally distributed. Consequently, Pearson correlation test was used to test the convergent validity and correlations of the TEMPS-M subscales and the other scales. The latter analysis was done using SPSS software v.22.

## Results

### Characteristics of the sample

Data was collected from 387 subjects from Lebanon (209 women, mean age_total sample_ = 35.39 years, SD = 14.21); 180 (46.5%) were married and 191 (53.2%) had a university level of education (Table 1).

**Table 1:** Characteristics of the participants (N = 387)

Variable	N (%)
*Gender*	
Male	172 (44.4%)
Female	209 (54.9%)
*Marital status*	
Single	180 (46.5%)
Married	180 (46.5%)
Widowed	11 (2.8%)
Divorced	13 (3.4%)
*Education*	
Secondary or less	168 (46.8%)
University	191 (53.2%)

### Confirmatory factor analysis

The five-factor solution of the TEMPS displayed a significant CFI of 0.94, TLI of 0.94 and a GFI of 0.95, but a poor RMSEA of 0.10 [90% CI 0.10, 0.11].

To improve this original model, which yielded relatively inadequate fit, we examined the modification index (MI) as recommended [54]. More specifically, the MI provide an estimate increase in the chi-square for each parameter if it were to be freed [55].

**Fig. 1:**
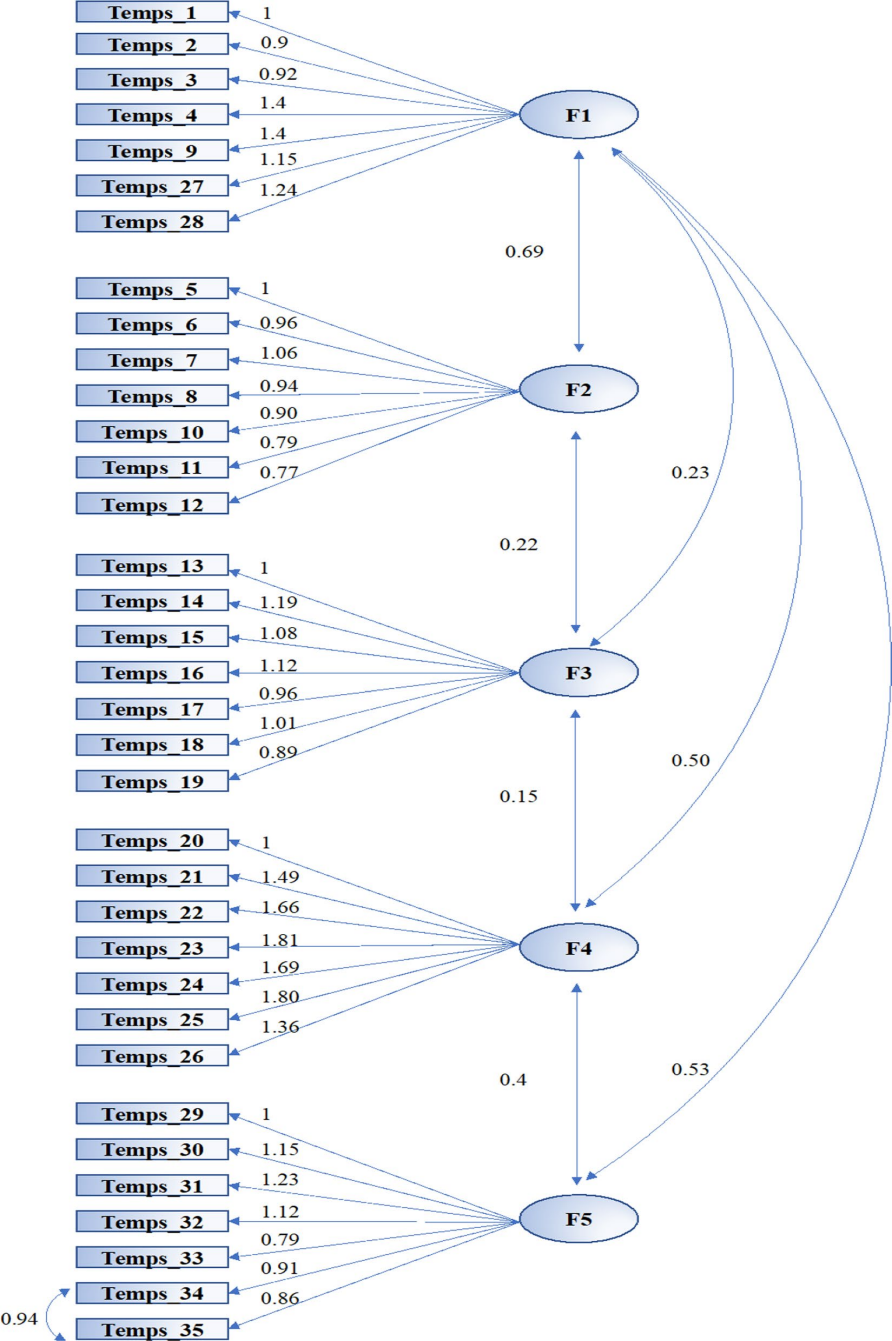
Standardized factor loadings and correlations between latent variables for five-factor model of the TEMPS (p < 0.001 for all loading factors). F1 = depressive, F2 = cyclothymic, F3 = hyperthymic, F4 = irritable, F5 = anxious

In the current study, the MI outlined a strong positive covariance (i.e., of 0.94) between items 34 and 35. Accordingly, a modified model considering this covariance was created. Firstly, compared the original model, the modified version demonstrated a lower chi-square (i.e., χ2 = 3078.348. and χ2 = 1369.111, respectively, with all p < 0.0001). As noted in previous studies [56] a low chi-square value relative to the degrees of freedom indicates a good model fit. Moreover, the second model demonstrated a significant CFI of 0.94, a TLI of 0.94, a GFI of 0.94 and a significantly decreased RMSEA of 0.06 [90% CI of RMSEA (0.058, 0.066)]. Standardized factor loadings and correlations between latent variables for five-factor model of the TEMPS can be found in Fig. 1. All five subscales achieved very good or good internal consistencies in the present study (depressive 0.78, cyclothymic 0.86, hyperthymic 0.83, irritable 0.87, and anxious 0.87).

### Measurement invariance between males and females

Next, we tested for gender invariance of the five-factor structure of the TEMPS-M scale. All indices suggested that configural, metric, and scalar invariance were supported across gender (Table 2). Consequently, we compared the temperament scores between genders using the Student t test. Men (*M* = 17.33, *SD* = 6.37) had significantly higher irritable temperament scores than women (*M* = 15.60, *SD* = 5.83), *t*(379) = 2.754, *p* = 0.006, *d* = 0.283. No significant difference was found between sexes for the other temperaments (Table 3). Older age was significantly associated with lower depressive (r = − 0.12; p = 0.019), cyclothymic (r = − 0.17; p = 0.001) and irritable (r = − 0.20; p < 0.001) temperaments, but was not associated with hyperthymic (r = − 0.04; p = 0.447) and anxious (r = − 0.03; p = 0.544) temperaments.

**Table 2:** Measurement invariance across sexes

Model	χ^2^	*df*	CFI	RMSEA	SRMR	GFI	TLI	Model Comparison	Δχ^2^	ΔCFI	ΔRMSEA	ΔSRMR	Δ*df*	*p*
Configural	1679.78	1098	.964	.051	.086	.981	.962							
Metric	1631.50	1128	.960	.054	.088	.980	.958	Configural versus metric	48.28	.004	.003	.002	30	0.018
Scalar	1665.33	549	.948	.062	.080	.980	.959	Metric versus scalar	33.84	.012	.008	.008	579	1

**Table 3:** Comparison of temperament scores between sexes

Temperament	Male	Female	t	*p*
Depressive	16.34 ± 5.09	15.91 ± 5.24	0.806	0.421
Cyclothymic	16.73 ± 5.72	17.03 ± 5.98	0.491	0.624
Hyperthymic	20.60 ± 5.72	19.49 ± 5.60	1.918	0.056
Irritable	17.33 ± 6.37	15.60 ± 5.83	2.754	0.006
Anxious	16.81 ± 6.30	16.37 ± 6.37	0.666	0.506

### Convergent validity and other correlations

Higher depressive, cyclothymic, irritable and anxious temperaments were significantly associated with more negative affect, detachment, antagonism, disinhibition and psychoticism. Moreover, all temperament dimensions were significantly associated with higher depression and anxiety scores. Finally, older age was significantly associated with lower depressive, cyclothymic and irritable temperaments (Table 4).

**Table 4:** Correlation of temperaments with other continuous variables

	1	2	3	4	5	6	7	8	9	10	11	12	13
1. Depressive temperament	1												
2. Cyclothymic temperament	.70***	1											
3. Hyperthymic temperament	.23***	.23***	1										
4. Irritable temperament	.51***	.41***	.15**	1									
5. Anxious temperament	.53***	.44***	.14**	.40***	1								
6. Negative affect	.36***	.30***	.05	.24***	.25***	1							
7. Detachment	.28***	.32***	− .01	.33***	.26***	.44***	1						
8. Antagonism	.29***	.33***	.07	.38***	.32***	.41***	.64***	1					
9. Disinhibition	.31***	.31***	.05	.40***	.20***	.50***	.52***	.56***	1				
10. Psychoticism	.37***	.36***	.07	.38***	.35***	.49***	.53***	.64***	.60***	1			
11. Anxiety	.36***	.29***	.13**	.23***	.46***	.29***	.24***	.30***	.24***	.38***	1		
12. Depression	.17**	.15**	.15**	.10*	.21***	.12*	.04	.05	.07	..12*	.49***	1	
13. Age	− .13*	− .18***	− .04	− .21***	− .02	− .01	− .11*	− .12*	− .13**	− .12*	.002	.05	1

*p < .05

**p < .0

***p < .001

## Discussion

The research aim of this study was to examine the psychometric properties of the Arabic version of 35-item TEMPS-M in a Lebanese community sample. We found that the Arabic TEMPS-M revealed good reliability (internal consistency). The five-factor model demonstrated adequate goodness of fit index. The factor structure between men and women was consistent, which maintained the stability of the factor covariance. In addition, evidence was provided for the convergent validity of the scale. We thus provide a shorter and still psychometrically robust scale to measure affective temperaments in Arab-speaking non-clinical populations. Making this scale available in the Arabic language will hopefully encourage Arab researchers to investigate this under-explored topic in their countries, and advance knowledge on how culture impacts the prevalence, development and course of temperament [57].

The validation of shorter versions of a psychological measure may reduce administration effort and time and enhance quality of responses; but it is not always beneficial if the measure does not preserve its validity, reliability, and factor structure [58]. The present study demonstrated that the internal consistency of the scale was good on the subscale level (Cronbach's α coefficients ranging from 0.78 to 0.87). In addition, while some of the previous validation works failed to maintain consistency with the original TEMPS-A factor structure [57], the exploratory factor analysis performed in our study showed that the 35-item version of the scale retained the five-factor structure of the original English version (depressive, cyclothymic, hyperthymic, irritable, anxious). These psychometric characteristics are similar to the Arabic TEMPS-A that have also been validated in a Lebanese population (five factors, alpha values per factor ranging from 0.76 to 0.88) [26]; and plead in favor of the validity of the Arabic TEMPS-M.

Convergent validity refers to whether a measured variable correlates with other measures of the same construct [59]. In the present study, the convergent validity of the Arabic TEMPS-M was tested in comparison to the five domains of personality dysfunction (i.e., Negative affect, Detachment, Antagonism, Disinhibition, Psychoticism); given that personality dimensions have been demonstrated to putatively overlap with temperament [60–62]. Our analyses showed positive correlations between depressive, cyclothymic, irritable, anxious temperaments and five maladaptive personality traits (negative affect, detachment, antagonism, disinhibition and psychoticism). Similar patterns of correlations have been previously noted in the previous studies [2, 60, 63]. For instance, the depressive temperament dimension was significantly related to dependent and avoidant personality traits [64, 65]. People with cyclothymic temperament has consistently been found to correlate with or borderline, histrionic and antisocial personality profiles [66–69]. These findings suggest temperamental origins of key personality constructs. Indeed, affective temperaments overlap with personality dimensions, and have even been suggested to differentiate into personality traits through gene-environment developmental processes [70]. Childhood temperament has also shown to provide predictive validity for later adulthood personality [71].

The five affective temperaments showed positive correlation with anxiety and depressions scores, highlighting certain temperament profiles as possible correlates for psychopathology symptoms [72]. Consistently, some evidence suggests a potential overlap between temperament and the development of psychopathology symptoms (e.g., [73, 74]). Temperamental characteristics were found to uniquely predict subsequent higher symptom levels for depression and anxiety [75].

Multi-group confirmatory factor analysis supported measurement invariance of the Arabic TEMPS-M between genders. Comparisons indicated higher irritable temperament scores in male participants compared to females. Previous research reported mixed results related to gender. Some studies documented gender effect for the cyclothymic, depressive, anxious and hyperthymic temperaments [76, 77], but not for the irritable temperament [31]. Other studies found, however, that females exhibited more depressive, anxious, cyclothymic [33], and less hyperthymic temperaments [78] than males. No studies explained exactly the underlying mechanisms of these differences, but it might mainly be due to complex factors such as sex-dependent neurobiology and genetic, hormonal and immune functions, as well as sex-environment interactions [79]. Other authors observed gender differences across cultures in temperament traits [80], which may explain our controversial findings and call for additional cross-cultural research. More specifically, in traditional cultures, perceived differences between men and women in general might be attributed to role requirements rather than to intrinsic differences in personality traits and temperaments. Thus, real differences in behavior might be seen everywhere, but would be attributed to roles rather than traits in traditional cultures (e.g. in individualistic, egalitarian countries, an act of kindness by a woman may be perceived as a free choice that must reflect on her personality. The same act by a woman in a collectivistic, traditional country might be dismissed as mere compliance with sex role norms).

### Limitations and future research directions

Certain limitations need to be considered when interpreting the current findings. Participants were recruited entirely online, with an overrepresentation of females and highly educated respondents; which may be source of selection bias. Also, individuals who do not have access to the Internet would have scored differently on the scales assessing temperament and psychopathology. No information regarding the history of mental disorders in participants was collected. The cross-sectional design limits the possibility of clarifying causality. Non-clinical community participants were only recruited to this study, limiting to some extent any generalization of our conclusions to clinical populations. Future studies need to validate the Arabic TEMPS-M in Arab-speaking patients with bipolar spectrum disorders (e.g., cyclothymic and/or bipolar disorder). Fourth, temperament profiles have proven to be shaped by cultural groups' norms, dynamics and practices, differing thus substantially across countries and cultural backgrounds [80]. However, we have only involved Lebanese participants in this validation work. Further research need to include participants from other Arab countries to test the validity of the scale across cultures and assess other psychometric properties of the scale (e.g., test–retest reliability, divergent validity).

## Conclusion

Our data suggest that the psychometric properties of the Arabic 35-item TEMPS-M are good, and preliminarily indicate that it can be used to evaluate affective temperament in a reliable manner in Arab speaking-populations. Given its relative shortness, the TEMPS-M is quicker, easier-to-use and less costly than the former Arabic version (i.e., the Arabic TEMPS-A), and can thus be included in any battery of assessments examining temperament for diagnosis or research use. However, we are aware that the Arabic TEMPS-M still requires further validation in clinical settings and adaptation in other Arab countries and cultural contexts to confirm its psychometric robustness.

## Data Availability

All data generated or analyzed during this study are not publicly available due to restrictions imposed by the ethics committee. The dataset supporting the conclusions is available upon request to the corresponding author (SH).
